# The Influence of Culture on the Cause, Diagnosis and Treatment of Serious Mental Illness (*Ufufunyana*): Perspectives of Traditional Health Practitioners in the Harry Gwala District, KwaZulu-Natal

**DOI:** 10.1007/s11013-024-09863-7

**Published:** 2024-06-23

**Authors:** Ntombifuthi P. Ngubane, Brenda Z. De Gama

**Affiliations:** Discipline of Clinical Anatomy, School of Laboratory Medicine and Medical Sciences, College of Health Sciences, University of KwaZulu-Natal, Westville Campus, Private Bag X54001, Durban, 4000 South Africa

**Keywords:** Ancestors, Mental illness, Traditional health practitioners, Cultural beliefs, Schizophrenia

## Abstract

Cultural beliefs influence the perceived cause, methods of diagnosis and treatment of mental illnesses. A qualitative study was conducted among traditional health practitioners (THPs) in the Harry Gwala District Municipality to further explore this influence. Purposive sampling assisted in the recruitment of 31 participants (9 males and 22 females). The four key themes this study investigated in relation to mental illness included its causes, methods of diagnosis, common symptoms observed and treatment approaches used by THPs, and the system of patient management. Culturally, mental illness was reported to be caused by witchcraft and an ancestral calling in this study. Mental illness was predominantly diagnosed by spiritual intervention which included divination through consultation with the ancestors, familial background, burning of incense which can also be part of communicating with the ancestors and through examining the patient. The common symptoms included aggression, hallucination and unresponsiveness. Prevalent modes of treatment included the use of a medicinal concoction and performing cultural rituals where ancestors and other spirits were assumed influential. The duration of the treatment process was dependent on guidance from the ancestors. Most causal aspects of mental illness from diagnosis to treatment seemed to be influenced by cultural beliefs and ancestors.

## Introduction

The use of traditional medicine also referred to as complementary and alternative medicine (CAM) dates back several decades and has been noted in different communities globally. This type of medicine is often referred to as an informal folk treatment which represents practices that are outside of conventional medicine (Budzak & Brankovic, 2022; Helha & Wang, 2022). According to the World Health Organisation (2019b), the term “complementary” describes approaches that may be used as augmentation to biomedical medicine, these include massage and meditation; whereas the term “alternative” describes methods such as herbal remedies which are used instead of conventional medicine. Despite traditional medicine lacking sufficient empirical investigation, it is estimated that approximately 80% of the global population uses this type of medicine for the treatment and management of ailments associated with mental, emotional, spiritual, physical and functional aspects (WHO, 2019b; Budzak & Brankovic, 2022; NCCIH, 2022). Traditional healing practices used by traditional health practitioners (THPs) play a significant role in the delivery of mental healthcare since receiving formal psychiatric treatment in low and middle-income countries remains a challenge for approximately 76-85% of individuals with serious mental illnesses (Nortje et al., 2016; WHO, 2021). The common factors that contribute to the inaccessibility to formal psychiatric healthcare and to the widespread use of traditional medicine in developing countries include, cost-effectiveness, dissatisfaction with biomedical, healthcare and the scarcity of mental healthcare professionals (Mpinga et al., 2013). Particularly, it has been reported in South Africa that there are approximately 1.5 psychiatrists and 2.5 psychologists per 100 000 individuals (WHO, 2019a; Padmanabhanunni et al., 2022). Traditional medicine then becomes the next available option due to its emphasis on holistic healthcare and conformity with spiritual and cultural beliefs and practices (Helha & Wang, 2022; Mpinga et al., 2013).

South Africa is a diverse country with its largest proportion constituted by 81.4% of the Black African population group which is divided into different tribes which are characterised by distinctive cultural beliefs and practices (StatsSA, 2022; Makhetha et al., 2024). Of the 11 official languages spoken by the different ethnic groups in South Africa, isiZulu (24.4%) and isiXhosa (16.3%) are the most commonly spoken languages with AmaZulu also being the largest of the ethnic groups (StatsSA, 2022). IsiZulu is dominant in the province of KwaZulu-Natal (KZN) and in some parts of Mpumalanga, Gauteng and Free State provinces, and isiXhosa is dominant in the Eastern Cape Province and the southwestern part of KZN (StatsSA, 2022). The effectiveness of traditional healing, as reported by its patients, is influenced by language as the THPs speak the languages native to their patients; thus, patients are able to accurately describe their symptoms to them compared to biomedical doctors (Ngubane & De Gama, 2023; Van der Watt et al., 2018). In many African communities, God (*uMvelinqangi* in Nguni languages) and the ancestors (*amadlozi* and *iinyanya* in isiZulu and isiXhosa, respectively) are the center of healing and are closely connected, with the ancestral spirits perceived as mediators between God and the living (Mokgobi, 2014; Zuma et al., 2016). The ancestral spirits are also reported to provide guidance to the THPs in diagnosing and treating different ailments, including mental illnesses (Zuma et al., 2016).

Culture is a significant factor that influences how various communities, particularly African communities, explain and perceive mental illness (Kleinman, 1977; Monama & Basson, 2017). Cultural background including values and belief systems, also play a pivotal role in the diagnosis and treatment of mental illnesses in the traditional healing community. Additionally, supernatural causal explanations which include bewitchment or witchcraft, sorcery, evil spirits and an ancestral calling (a gift or a calling to become a THP) are believed to be the cause of mental illness, particularly psychosis, in this community in Africa (Zabow, 2007; Al-Krenawi, 2019; van der Zeijst et al., 2021a, 2021b, 2022). However, in some instances, the pathological potential of the ancestral calling in mental illness may be argued as cultural beliefs vary between different communities (van der Zeijst et al., 2021a, 2021b). Nonetheless, psychotic and mood-related symptoms have been reported to be common in individuals with an ancestral calling (van der Zeijst et al., 2022). Usually the spirits of the ancestors, particularly if they have been neglected or are not remembered and properly treated, can become spirit agents that cause mental illness, which is commonly associated with “bad spirits” that could originate from rocks, lakes and rivers (Verginer & Juen, 2019). Additionally, in the traditional healing context, schizophrenic symptoms are described as *ufufunyana/amafufunyana* which is also defined as a culture-bound syndrome and is further used in explaining “aberrant behavioral and psychological phenomena” (Molot, 2017; Volkan, 2021). *Ufufunyana* is reported to be caused by witchcraft leading to evil spirit possession (Molot, 2017). This results in different views on the cause of mental illness between biomedical and traditional healing. There also exists the notion in biomedical healing that the cause of mental illness is biologically-oriented while African traditional healing emphasizes supernatural or religious-magical views (Teferra & Shibre, 2012).

In biomedical healing, mental illness is defined as a health problem that affects the feelings, thoughts and behaviour of an individual which may result in the person’s quality of life being reduced (Ngobe et al., 2021). According to the World Health Organisation (2022), mental disorders are depicted by the disturbance of behaviour, cognition and emotional regulation of an individual. Numerous factors that contribute to mental health problems in South Africa include use substances, unemployment, poverty, diseases such as HIV/AIDS, political violence and transgenerational trauma as a result of European colonialisation (Madzhie et al., 2014; Gone & Kirmayer, 2020; Shange & Ross, 2022). The Diagnostic and Statistical Manual of Mental Disorders (DSM-5) manual is used in biomedical healthcare to diagnose mental illness based on the defining symptoms of each disorder. Also, treatment options are dependent on the type of illness and include; medications (such as antipsychotic drugs), psychotherapy, brain-stimulation and substance misuse treatments (Mayo clinic, n.d.). Traditional healing differs from biomedical healing as there is emphasis on the consultation with ancestors and the prescription of a medicinal concoction as common methods used by THPs for the diagnosis and treatment or management of mental illness (Shange & Ross, 2022; Sorsdahl et al., 2010). In cases of an ancestral calling, *ukuthwasa* (training to become a THP) may be the recommended form of treatment intervention (van der Zeijst et al., 2021a, 2021b).

This paper aimed to qualitatively obtain insights from the THPs’ perspective into how culture influences the explanation of mental disorders; viz. their cause, methods of diagnoses and treatment approaches used in the traditional healing practice. It also aimed to identify the common symptoms observed by the THPs in mentally ill patients and document the system used by the THPs to manage their patients. Further, this study had a specific focus on schizophrenia as symptoms of this disorder are said to be caused by a calling to become a THP (Sorsdahl et al., 2010) and are associated with conditions involving ancestors and/or other spirits and sorcery or bewitchment in the African healing setting.

## Material and Methods

### Research Design

The larger study included quantitative (Ngubane & De Gama, 2023) and qualitative components; however, the current article focuses only on the qualitative dimension of the research. With the use of focus group discussions, the phenomenology theoretical perspective was adopted in order to investigate the dynamics of the collective beliefs and perspectives of the THPs on mental illness. With this theory, data was analysed by allowing meanings to emanate from the in-depth semi-structured group interviews that were conducted. The thematic analysis method was adopted to extract and synthesize key information among the THPs using codes and themes.

### Recruitment

**Fig. 1:**
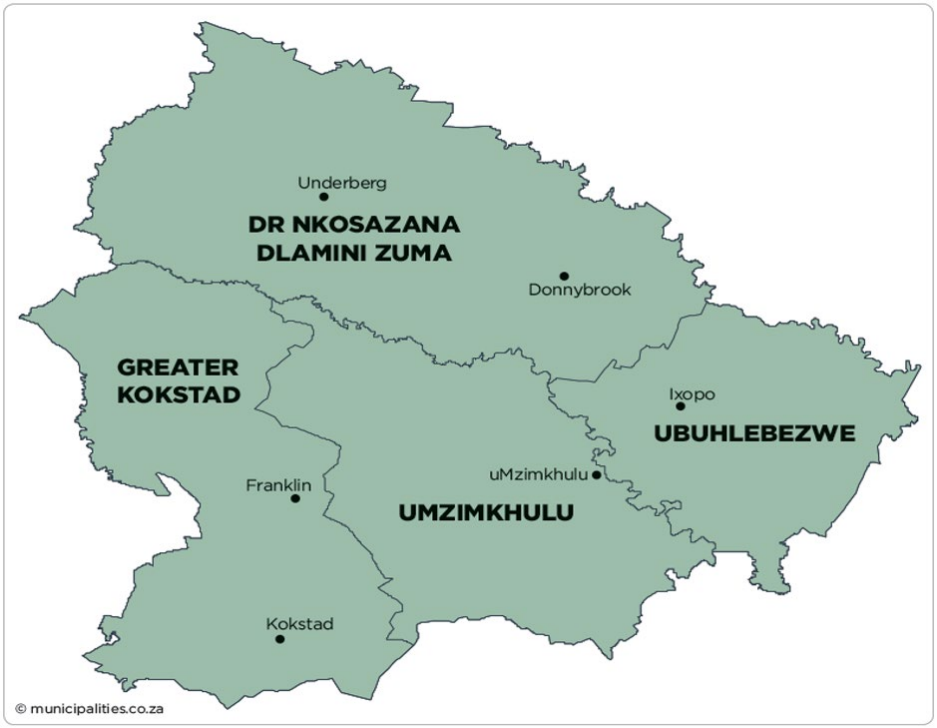
Local Municipalities of the Harry Gwala district municipality (Adapted from https://municipalities.co.za)

This study was conducted in the Greater Kokstad (GK), uMzimkhulu (Mz), uBuhlebezwe (Bu) and Dr. Nkosazana Dlamini Zuma municipalities (NDZ) which are local municipalities of the Harry Gwala District Municipality (HGDM) located southwest of the KwaZulu-Natal Province (Fig. 1).

There were four focus groups as per the local municipalities with participants ranging from 5-10 per group. A group of 8 THPs were interviewed in Greater Kokstad Municipality, 8 THPs in uMzimkhulu Municipality, 10 in uBuhlebezwe Municipality and 5 were interviewed in Dr. Nkosazana Dlamini Zuma Municipality. All focus group discussions were conducted in February 2022. Purposive Sampling was used as the participants were selected from the THPs who had participated in the quantitative component of the research project and had indicated possession of knowledge on methods of diagnosis and treatment of mental disorders. In the previous study by Ngubane and De Gama (2023), 100 questionnaires were administered to randomly selected THPs from the HGDM as part of the quantitative investigation which aimed to add on the knowledge of the socio-demographic profiles of the THPs in this district, their knowledge and methods of diagnosis and treatment of mental disorders.

### The Participants

A total of 31 THPs (71% female; 29% males) participated in this study. The age range of the participants was between 30 and 67 years with a mean age of 47 years. A total of 52% THPs classified as a diviner (*Isangoma* in IsiZulu), 6% as an herbalist (*Inyanga* in IsiZulu), 6% as a faith healer (*Umthandazi* in IsiZulu) and 35% classified as a combination of these classifications. The years of experience of the THPs in the traditional healing practice ranged between 3 and 33 years with a mean range of 12 years (Table 1). The sample consisted of THPs from the Zulu and Xhosa ethnic groups of South Africa, who communicated in both IsiZulu and IsiXhosa languages. The THPs who participated in this study were registered under the Harry Gwala District Traditional Health Practitioners organization.

**Table 1:** Demographics of the participants as per focus group discussions in each local municipality

Local municipality	Participant No.	Age (years)	Gender	Classification	Experience (years)
Greater Kokstad	1	40	Female	Combination	17
	2	54	Female	Combination	15
	3	43	Female	Combination	4
	4	47	Female	Isangoma	10
	5	45	Female	Combination	20
	6	34	Female	Combination	8
	7	47	Female	Combination	12
	8	30	Male	Isangoma	7
Buhlebezwe	1	57	Female	Isangoma	14
	2	40	Male	Combination	18
	3	53	Female	Isangoma	10
	4	42	Female	Isangoma	7
	5	36	Female	Isangoma	18
	6	49	Female	Isangoma	8
	7	62	Male	Combination	33
	8	47	Male	Umthandazi	7
	9	64	Female	Umthandazi	24
	10	30	Female	Isangoma	4
uMzimkhulu	1	51	Female	Isangoma	16
	2	30	Female	Isangoma	4
	3	38	Male	Isangoma	7
	4	56	Female	Isangoma	8
	5	59	Female	Isangoma	13
	6	46	Female	Combination	10
	7	58	Male	Inyanga	16
	8	63	Female	Combination	3
Dr. Nkosazana Zuma	1	32	Male	Isangoma	14
	2	54	Female	Isangoma	4
	3	59	Female	Combination	29
	4	67	Male	Inyanga	5
	5	31	Male	Isangoma	9

### Focus Groups

The focus group discussions were guided by semi-structured questions from a self-developed interview sheet on the cause of mental illness, symptoms and the traditional methods of diagnosis and treatment of the different kinds of mental disorders or illnesses (Appendix). Each session was conducted between 40 and 90 min. A pre-test of the interview schedule was conducted with two THPs (which were excluded from the study) prior to its use in the actual focus group discussions in order to ensure reliability and validity of the questions. The group discussions, were co-facilitated by both authors and each participant was provided with an equal chance to contribute to the discussion. THPs participating in the group discussions were not asked or pressured to confirm contributions from one THP. All sessions were recorded and were supplemented by handwritten notes.

### Inclusion and Exclusion Criteria

Registered THPs in the Harry Gwala District Municipality who were invited to participate in this study based on their responses from the questionnaire administered prior to commencement of the qualitative component of the study were included. THPs who had not participated in the quantitative component of the study were excluded from the focus group discussions.

### Data Analysis

All focus group sessions were recorded and later transcribed verbatim in the language that they were conducted in. Key information from the transcriptions was translated into English by both authors independently in order to ensure consistency and accuracy. The six phases of thematic analysis as described by Braun and Clarke (2006) were used in order to identify meanings and patterns in the accounts of the participants. The authors independently coded each transcript manually. The patterns and trends which emerged from the data were then classified into themes, and areas of inconsistency and disagreement were discussed by both authors until consensus on the appropriate coding was reached. Both authors also reviewed and refined these classifications for proper definition of the themes. The refined themes which emerged are presented in the results section. The narratives of the participants are anonymously reported.

### Ethical Consideration

Ethical approval for this study was obtained from the Human Social Sciences Research Ethics Committee of the University of KwaZulu-Natal (HSSREC/00002944/2021) prior to data collection. All participants were fully informed about the aim and significance of the study prior to its commencement and an informed consent form was signed prior to participation.

## Results

Of the 31 THPs that participated in this study, the majority were from the uBuhlebezwe Municipality [32%; n = 10]. The majority of the THPs were females [71%; n = 22], and 52% (n = 16) of the 31 THPs classified as *Isangoma* (Table 1). The age range of the participants was 30–67 years and their years of experience ranged from 3 to 33 years (Table 1).

The findings are presented as per the key themes of this study; viz. (1) cause of mental illness, (2) traditional methods of diagnosis, (3) identification of common symptoms and treatment methods of mental illness among patients, and (4) the system of patient management. The sub-themes that emanated under the cause of mental illness included witchcraft and ancestral calling. Divination through consultation with the ancestors, family background, burning of incense and examining the patient were the sub-themes that emerged under the methods of diagnosis. Aggression, hallucination, inability to speak and unresponsiveness emanated under the common symptoms observed and the sub-themes that emerged under the methods of treatment included the use of medicinal preparations, guidance from ancestors on treatment approach to use and performing of cultural rituals. Record keeping and referral to biomedical healthcare facilities were the sub-themes that emerged under the system of patient management. A total of 31 THPs participated in the focus group discussions and individual responses were voluntarily offered with no pressure for other THPs to confirm this narrative. The comments offered by the THPs in each key theme are quoted below. Narratives from the THPs have been reported as n = x, and the following abbreviations were used for responses from each local municipality: Greater Kokstad (GK), uMzimkhulu (Mz), uBuhlebezwe (Bu) and Dr. Nkosazana Dlamini Zuma (NDZ) to offer information of how widely a specific response was mentioned. The total number of responses received for each narrative was presented as n^total^.

### Cause of Mental Illness

The prevalent causes of mental illness, particularly *ufufunyana* (which may be interpreted as schizophrenia and/or the possession by evil spirits), across all four local municipalities included an ancestral calling (or issues relating to ancestors) [n^total^ = 14: n = 4 (GK), n = 5 (Mz), n = 3 (Bu), n = 2 (NDZ)], witchcraft (which in some cases included bad spirits, i.e. *isilwane* in IsiZulu) [n^total^ = 13: n = 5 (GK), n = 3 (Mz), n = 5 (Bu)] and the use of substances [n^total^ = 7: n = 4 (GK), n = 2 (Bu), n = 1 (NDZ)]. In one case [n = 1 (GK)] *ufufunyana* was not classified as a mental disorder but the cause of mental illness.

My patients were suffering from ufufunyana. The first one (of the aforementioned patients) ubehlushwa abantu abadala (had an ancestral calling), this started when they were young and it was ignored in their family until the ancestors took away their sanity. (P6, Greater Kokstad Municipality)

Most of the patients I have treated had ufufunyana which was inflicted. The cause of mental illness in 2 of the patients was isilwane sihamba nalo ufufunyana (witchcraft inflicted ufufunyana). (P2, Greater Kokstad Municipality)

Of the 4 patients that I have treated, drugs were the cause of mental illness in 2. (P1, Greater Kokstad Municipality)

A person becomes mentally ill because of imimoya yokuthi uthakathiwe (bad spirits inflicted by witchcraft/bewitchment), or they are just crazy and some mental illness has to do with ancestors. In some cases, a person can have ancestors fighting for them, maybe that person is using a surname that is not theirs and the ancestors from their true surname those from the current surname end up fighting over them. (P3, uBuhlebezwe Municipality)

Other causes that were reported were confusion [n^total^ = 3: n = 1 (GK), n = 1 (Bu), n = 1 (NDZ)] and heredity [n = 1 (NDZ)].

In some cases, it is just confusion, in some it is heredity and in some it is related to ancestors. (P2, Dr. Nkosazana Dlamini Zuma Municipality)

Defaulting from HIV treatment [n = 4 (Mz)] and stress [n^total^ = 4: n = 2 (Mz), n = 2 (NDZ)] were also reported as causes of mental illness.

I have diagnosed ufufunyana caused by not accepting the spiritual gifts that your ancestors give to you. In other patients, mental illness is caused by too much stress which then leads to depression. Others default from their HIV treatment and then say they have an ancestral calling when that is not the case. (P3, uMzimkhulu Municipality)

I have diagnosed ufufunyana and as I was questioning the patient's parents I discovered that the patient defaulted from their HIV treatment. It happens that when you default from HIV treatment you become mentally ill. Sometimes mental illness is due to umsamo ongalunganga (when the sacred place or alter where there is interconnectedness with the ancestors is not in a good state), sometimes ufufunyana is due to izinto zabantu (bewitchment) and ancestors. (P5, uMzimkhulu Municipality)

### Traditional Methods of Diagnosis

The common method of diagnosis across all 4 local municipalities was divination through consultation with the ancestors [n^total^ = 10: n = 3 (GK), n = 1 (Mz), n = 3 (Bu), n = 3 (NDZ)].

You first consult with the ancestors and that is where you will get the answers and how you need to help your patient. (P3, Greater Kokstad Municipality)

I consult with ancestors as the patient comes in and they advise on what is wrong with the patient and how I should treat them. (P2, uMzimkhulu Municipality)

Seeking information from the family members or from the individuals accompanying the patient [n^total^ = 3: n = 1 (GK), n = 2 (Mz)] was also reported as a method of diagnosis.

You first find out from the people who bring in the patient what is wrong with the patient. You then consult with the ancestors and they will give you the diagnosis. (P2, Greater Kokstad Municipality)

Other methods used in uBuhlebezwe included burning of incense (*impepho* in IsiZulu) [n = 1 (Bu)].

The first thing we do as THPs when a patient is brought in is burn incense and then consult with the ancestors. (P2, uBuhlebezwe Municipality)

A method that can only be done by an experienced THP was deducing and confirming the diagnosis from just examining the patient [n = 1(NDZ)]. This examination was not elaborated further by the THP.

There are ways to examine the patient to better understand the diagnosis. (P1, Dr. Nkosazana Dlamini Zuma Municipality – 14 years of experience)

### Identification of Common Symptoms and Treatment Methods of Mental Illness Among Patients

#### Common Symptoms

Aggression [n^total^ = 11: n = 5 (GK), n = 4 (Mz), n = 2 (Bu)], hallucination [n^total^ = 8: n = 4 (GK), n = 1 (Mz), n = 3 (NDZ)], inability to speak [n^total^ = 5: n = 1 (GK), n = 3 (Bu), n = 1 (NDZ)], being incoherent [n^total^ = 4: n = 3 (Bu), n = 1 (NDZ)] and unresponsiveness [n = 2 (Mz)] were commonly observed in patients with mental illness. Other symptoms included not eating [n^total^ = 3: n = 1 (Mz), n = 2 (Bu)], inability to walk [n = 1(GK)] and lack of sleep [n = 1 (Mz)].

One who had ufufunyana was chasing and killing chickens and also said they were seeing some people. Another one who had ufufunyana saw people who were chasing them. (P3, Greater Kokstad Municipality)

One child could not walk and speak due to the illness. (P4, Greater Kokstad Municipality)

The patient was tied up and in pain. They were said to be aggressive, they tore their clothes, destroyed everything in the house, they were not eating or sleeping. (P5, uMzimkhulu Municipality)

Other symptoms identified included hysterical screams [n^total^ = 6: n = 1 (GK), n = 1 (Mz), n = 3 (Bu), n = 1 (NDZ)], seizures [n^total^ = 6: n = 4 (GK), n = 2 (Bu)], being fearful [n = 2 (Bu)] and forgetfulness [n = 1 (Bu)].

In some cases, it can happen that a person speaks things that do not make sense and they are forgetful. Also, it can happen that when a patient has bad spirits they will have fear and this may cause them to scream. (P2, uBuhlebezwe Municipality)

A person with ufufunyana may just scream for no reason and does not talk a lot. And then there is a person who is just crazy and talks a lot, even things that do not make sense. (P1, Dr. Nkosazana Dlamini Zuma Municipality)

#### Treatment Methods

Mental illnesses were reported to be commonly treated using *umuthi* or a medicinal concoction [n^total^ = 16: n = 3 (GK), n = 5 (Mz), n = 5 (Bu), n = 3 (NDZ)]. The predominant modes of administration were inhalation [n^total^ = 7: n = 2 (GK), n = 4 (Bu), n = 1 (NDZ)], drinking [n^total^ = 7: n = 3 (GK), n = 2 (Mz), n = 2 (Bu)], putting or squeezing into the nose [n^total^ = 4: n = 1 (GK), n = 1 (Mz), n = 2 (Bu)], *ukuchatha* (enema) [n^total^ = 7: n = 1 (GK), n = 5 (Bu), n = 1 (NDZ)], *ukuphalazisa* (induced vomiting) [n^total^ = 4: n = 1 (Mz), n = 2 (Bu), n = 1 (NDZ)] and steaming [n^total^ = 3: n = 2 (Bu), n = 1 (NDZ)].

When it comes to ufufunyana we use traditional medicines which we get from the forest. (P2, Greater Kokstad Municipality)

Medicinal concoctions are used to treat mental illness and these are administered through inhalation, ukuphalaza (induced vomiting) and enema. (P1, uBuhlebezwe Municipality)

Usually for mentally ill patients, medication is administered through inhalation to try and calm the patient, steaming, induced vomiting and enema are also used. (P5, Dr. Nkosazana Dlamini Zuma Municipality)

Cleansing the patient was reported to be the first step to treatment in some cases [n^total^ = 3: n = 1 (GK), n = 2(Bu)] and a patient could be treated by performing their required cultural rituals in other cases [n^total^ = 4: n = 2 (GK), n = 2 (Bu)].

You first need to cleanse the patient before you can start with treatment. (P1, Greater Kokstad Municipality)

When the illness has to do with ancestors, appropriate rituals must be done. (P6, Greater Kokstad Municipality)

Guidance from the ancestors on the best and most appropriate method of treatment was required by some of the THPs [n^total^ = 6: n = 2 (GK), n = 3 (Bu), n = 1 (NDZ)] prior to treating their patients.

Usually we are guided by the ancestors we work for. But usually you have to give the patient with mental illness something for inhalation to ease the illness. (P5, Dr. Nkosazana Dlamini Zuma Municipality)

In some cases [n = 3 (Mz)], the treatment method was dependent on the symptoms presented by the patients.

As there are different symptoms, the modes of treatment are also different. In some cases, a mentally ill person does not require to be treated with pills but with traditional medicines (ubulawu) when the illness has to do with ancestors. A person with umdlenyane (form of ufufunyana) is treated using a medicinal concoction. (P7, uMzimkhulu Municipality)

### The System of Patient Management

Keeping patient records [n^total^ = 15: n = 5 (GK), n = 7 (Mz), n = 2 (Bu), n = 1 (NDZ)] and conducting follow-ups [n^total^ = 6: n = 2 (GK), n = 2 (Mz), n = 1 (Bu), n = 1 (NDZ)] was reported across all 4 local municipalities. Records included patient’s personal details and history which included information on prior illnesses and treatment, which also included the history of any cultural practice or rituals that have been conducted before to or for the patient.

It is important to keep records of your patients and to have their medical history. (P1, Greater Kokstad Municipality)

We also need to know of traditional rituals that have been performed for or to the patient. (P6, Greater Kokstad Municipality)

We do have books where we keep all records of our patients (their names, surnames, contact details and addresses). We also keep medical and treatment history of the patients. We also do follow-up on our patients and find out how the treatment process is going. (P2, uMzimkhulu Municipality)

It is important that when a patient comes to you, their name, surname and contact details are recorded. (P5, Dr. Nkosazana Dlamini Zuma Municipality)

Record keeping was reported to ensure ease of tracking the patient’s illness and medical history or records also assisted in providing clues for diagnosing and treating the current presented illness.

We do have books where we keep all records of our patients. We also keep medical and treatment history of the patients. We also do follow-up on our patients and find out how the treatment process is going. (P1, uMzimkhulu Municipality)

Record keeping was stated to be mandatory as per regulations by the Department of Health.

According to the regulations from the department of health, we have to have registers where we record details of patients that come to see us for each day. (P1, uBuhlebezwe Municipality)

THPs also reflected that they referred patients to biomedical institutions [n^total^ = 9: n = 2 (GK), n = 5 (Mz), n = 1 (Bu), n = 1 (NDZ)]. The common reason for referring patients to biomedical institutions across the 4 local municipalities was when traditional medicine approaches were ineffective and the THPs were unable to assist the patient. In Kokstad, pre-existing medical conditions such as diabetes and HIV were reported to be some of the illnesses which required patients to also use allopathic medicine prior to receiving traditional medicines.

A patient with diabetes cannot stop taking their treatment when they are being treated with traditional medicines, same goes for a person with HIV. So, these ailments have to be attended to with the aid of western medicine prior to treatment with traditional medicines because umuthi is strong. (P2, Greater Kokstad Municipality)

THPs maintained that their treatment approaches were strong and required that their patients be in good health prior to receiving treatment from them; hence, it was preferable that patients first conduct a medical checkup and if they default from their western medication or if the THPs cannot treat them due to various reasons they are referred to biomedical facilities.

When a person defaults from their HIV treatment you need to send or refer them to a clinic where they will advise what needs to be done. We also refer a person with ufufunyana to the clinic, especially if they do not eat, they need drips for energy because we cannot use umuthi on a weak person. Sometimes we take our patients to clinics to have them checked for underlying illnesses before we can use umuthi on them. (P1, uMzimkhulu Municipality)

If I cannot treat the person I refer them to the clinic where they get treated. (P2, uMzimkhulu Municipality)

I once had a mentally ill patient who would have seizures that were unstoppable and no matter what I did or gave to him I just could not help. So, what helped was rushing him to the nearest hospital or clinic. (P2, uBuhlebezwe Municipality)

In this day and age there are ailments that we as THPs cannot treat. So even if we try to help a patient whichever way possible, there also has to be an intervention from western medicine for the patient to be treated. (P5, Dr. Nkosazana Dlamini Zuma Municipality).

## Discussion

The psychiatric fraternity has been faced by culture-bound syndromes since past centuries, and these conditions are perceived to not fit accurately to the standard psychiatric classifications even though they are also said to be similar to psychiatric disorders in biomedical psychiatry (Wessels, 1985). One of these culture-bound syndromes is *ufufunyana/amafufunyana* which is defined as a possession by evil spirits through sorcery, with its sufferers being described as having hysterical psychoses (Wessel, 1985; Zabow, 2007; Molot, 2017). In IsiZulu/IsiXhosa, schizophrenia may be referred to as *amafufunyana* due to the alignment of symptoms between the two conditions, resulting in a complexity as they possess both a supernatural causation and biomedical-defined explanations (Molot, 2017). Discrepancies exist in the description of symptoms observed in patients diagnosed with *ufufunyana*; as its causation is usually reported differently depending on how respondents from different studies understand this condition (Parle, 2003). In the current study, *ufufunyana* was used by the THPs as a translation for schizophrenia or schizophrenic symptoms which is influenced by the specific cultural beliefs on ancestral calling and witchcraft thus affirming *ufufunyana* as a culture bound syndrome. However, *ufufunyana* was also indicated as one of the causes of mental illness in this study, agreeing with Fabrega (1991) in reporting u*fufunyana* to cause psychiatric illness. Zabow (2007) and Molot (2017) further attested to this in their definition of *ufufunyana* as a possession by evil spirits and the possession by these spirits which resulted in mental disturbance.

Culture-bound syndromes are described as a broad rubric that encompasses certain behavioral, affective and cognitive manifestations seen in specific cultures (Balhara, 2011). Although Balhara (2011) advocates for the relabeling of some of the culture-bound syndromes to functional somatic syndromes, the authors still adopt that the interpretation of *ufufunyana* as a culture-bound syndrome is accurate as per the findings of the current study. Additionally, even though there is an overlap of symptoms between *ufufunyana* and schizophrenia, due to the completely different views between African healing and biomedical healthcare on the causation of the two conditions, *ufufunyana* cannot be fully interpreted as a psychiatric condition such as schizophrenia. With the varying causal factors between these two conditions, the methods of diagnosis and treatment are inevitably unique in each fraternity, with all methods in African healing encompassed by cultural beliefs. For this reason, it seems more accurate for *ufufunyana* to be framed as a culture-bound syndrome since the manifestations associated with this condition are specific to the Zulu and Xhosa cultures.

The prevalent causes of mental illness in this study included the use of substances, witchcraft and an ancestral calling. The possession by evil or bad spirits, bewitchment or witchcraft, having an ancestral calling and use of substances as the cause of mental illness were also reported by other authors from Ethiopia and Uganda in Africa (Teferra & Shibre, 2012; Verginer & Juen, 2019) and from Mpumalanga, Gauteng and KwaZulu-Natal provinces in South Africa (Kometsi et al., 2020; Ngobe et al., 2021; Shange & Ross, 2022; Sorsdahl et al., 2010). Other causes in this study were stress, trauma, confusion, heredity and defaulting from HIV treatment, of which stress and trauma were consistent with reports by Teferra and Shibre (2012) and Kometsi et al. (2020). Mental illness inherited from parents by children was also reported by Teferra and Shibre (2012) and Shange and Ross (2022) corroborating perspectives from the NDZ municipality in this study. The involvement of HIV in the etiology of mental illness was also reported by Kometsi et al. (2020); however, it was the positive status of this illness that led to depression and schizophrenia in their study, though defaulting from HIV treatment causing mental illness was not included.

The prevalent methods of diagnosis included divination through consultation with ancestors (spiritual intervention), seeking information from the family members or the individuals accompanying the patient, burning of incense and examining the patient which were consistent with the prevalent methods of diagnosis reported by Kpobi et al. (2019) and Shange and Ross (2022), but consultation with ancestors was accompanied by throwing of bones in the study by Shange and Ross (2022). Burning of incense (*impepho*) can be used in the communication with ancestors and can be also used to calm patients or the evil spirits possessing the patients (Belani et al., 2022; Ntshangase, 2012). Some of the THPs in the study by Sorsdahl et al. (2010) also maintained that consulting with ancestors aided in the diagnosis of mental illness. Aggression, hallucination, unresponsiveness and also being incoherent were prevalent symptoms in mentally ill patients in this study. Other symptoms included the inability to walk, inability to talk, visual hallucinations, lack of sleep, not eating, stress, calling out names of people unseen by others, auditory hallucinations, being fearful, screams, seizures, forgetfulness and alogia. Hallucinations and delusions were also reported by Ngobe et al. (2021). Hallucinations were reported by van der Zeijst et al. (2020) as the prevalent symptom of the psychotic symptoms among apprentice THPs, with depressive symptoms prevalent under mood-related symptoms. In some cases, the evil spirits could switch the presented symptoms in a patient in order to confuse the healer and keep them from understanding the cause of the illness, this then delayed the treatment process (Verginer & Juen, 2019).

The prevalent method for treatment of mental illness in this study was the use of a medicinal concoction which is prepared using herbs or herbal plants. The details on the medicinal plants used in the concoction were not shared as this knowledge was regarded as sacred by the THPs since it was gifted to them by their ancestors, which is consistent with the findings by Sorsdahl et al. (2010). The THPs recommended that the authors visit the *muthi* market (which may also refer to the Zulu pharmacy) in order to obtain more information on the medicinal plants used for mental illness. The commonly used modes of administration of these herbal remedies were inhalation, drinking, steaming, enema (*ukuchatha*) and induced vomiting (*ukuphalazisa*). Giving patients a medicinal concoction for drinking and bathing was also reported by Sorsdahl et al. (2010). The THPs in this study maintained that cleansing the patient prior to treatment is significant and that sometimes a patient can be treated from their illness when cultural rituals have been kept for them. When considering conditions associated to an ancestral calling and other ancestral related issues, the THPs maintained that a patient can only be treated once they have accepted their calling and once all cultural rituals have been done for them. This was consistent with reports by Ngobe et al. (2021). It was also reported in this study that the treatment method is dependent on the symptoms presented by the patients and that guidance from the ancestors also plays a significant role towards determining how patients should be treated. Western medicine, particularly the pharmacological interventions only manage the symptoms of mental illness and do not attend to the root cause of the illness (Verginer & Juen, 2019), which is one of the factors contributing to the dissatisfaction with biomedical medicine. The THPs in this study maintained that the duration of treatment was dependent on the improvement of the symptoms presented by the patient and mainly on the guidance from the ancestors, hence the treatment process could last for days, months or years depending on what is communicated by the ancestors to the THP about the patient. The THPs reported that they follow-up on the patient’s progress if the patient is not staying with them for the duration of the treatment process and the records of each patient are maintained as per the regulations by the Department of Health. If the THPs fail to treat the patient using all their traditional strategies, they refer them to biomedical facilities such as clinics and hospitals thereafter.

Based on the findings from this study, there is some possibility of a fully functional collaboration between the African healing and biomedical medicine in psychiatry. Aspiration for this is drawn from the apparent collaboration noted when the THPs refer their patients to biomedical facilities for pre-existing illnesses such as diabetes and HIV prior to the commencement of their treatment methods. However, a question remains, if the THPs acknowledge the role of biomedical medicine for the management of other ailments, why is this not the case for mental illness? Perhaps the education and training of THPs regarding mental disorders, their diagnosis and treatment methods used in Western psychiatry could pave a way for a collaboration in psychiatry. For example, in a pilot study by Mbeh et al. (2010), THPs were trained to provide education and appropriate healthcare to diabetic patients in Cameroon. The findings of their study suggested that through this initiative, the THPs were enthusiastic about a collaboration with biomedical healthcare for the management of diabetes. This current study also noted that in other cases the THPs only refer their patients for biomedical healthcare after their treatment attempts have failed. Considering that the duration of their treatment is not clear, this might increase the risk of the illness worsening before the patient receives biomedical healthcare. Thus, further questions arise as to whether the referred patients were correctly diagnosed and treated? Furthermore, concerns also arise on whether the guidance from ancestors regarding the methods of diagnosis and treatment could be misleading in some instances.

## Conclusion, limitations and recommendations

It is evident from the study that the traditional healing practice is significantly influenced by ancestral spirits or the belief in these spirits. Ancestors and cultural beliefs also influence the beliefs regarding the etiology of mental illness and the methods used by the THPs to diagnose and treat mental illness. In order to further explore the extent of the influence of culture and ancestors in mental illness, it is recommended that future studies should also explore the perspectives of the patients and their caregivers. Further engagement with biomedical practitioners on their views of traditional healing practices for mental illness should be considered to offer a holistic understanding of healing within the mental health space in South Africa. Apart from referrals, it was evident that there was a working relationship or collaboration between traditional and biomedical medicine treatment approaches. This was validated by the THPs when they recommend patients to seek a medical check-up at the biomedical facilities prior to seeking traditional treatment. In addition, they also refer patients who have other ailments or pre-existing co-morbidities to biomedical facilities. However, this form of collaboration seemed one-sided as it was the THPs who referred their patients to biomedical facilities and no reports of referred patients from biomedical healthcare providers to the traditional healing practice were documented.

The major limitation of this study was the inability to obtain extensive details on the methods of diagnosis and treatment used by the THPs as almost all aspects of these were associated with the guidance from the ancestors and in other instances some information was described as sensitive and sacred, thus could not be shared with the investigators. It then becomes a challenge to attempt to find common ground between psychiatry between traditional healing and biomedical medicine. Perhaps the symptoms identified by the THPs could be a starting point in attempting to bridge the gap in the knowledge, diagnostic and management approaches between traditional and biomedical medicines. Although in most cases the causal factors for mental illness in African healing are linked with supernatural explanations, perhaps if the two healthcare entities allow themselves to learn from each other, a collaboration would be possible without one entity undermining the other based on the existing discrepancies.

## Appendix: The Role of Indigenous Healers and the Effect of Indigenous Medicine in the Treatment of the Negative Symptoms of Schizophrenia in KwaZulu Natal

**DATE: _______________ TIME: _____________ PARTICIPANT NO: ________**

**VENUE: ___________________________________________________________________**

**QUESTIONNAIRE PARTICIPANT NO: __________**

## Interview Questions

### History of Practice in Mental Health Disorders

1. - Since the start of your practice, how many patients have you diagnosed with mental health disorders?
  - How often do you diagnose individuals suffering from mental disorders?
  - From your experience, what is the cause of these mental health disorders?
  - How many patients are presently being treated?
    i. What is the common age group?
    ii. What is the more common gender profile of these patients?
    iii. Do you verify if the patient has accessed treatment before?
      Yes □ No □
    iv. What kind of treatment do patients usually access? (Western or Traditional)
    v. Do you keep a record of the treatment the patient has been exposed to previously?
      Yes □ No □
    vi. What other patient history do you require to be shared with you?

### Nature of disorders

2. What were the common symptoms noted?
3. Methods of diagnosis:
  - Is there a way you categorize mental health disorders based on the symptoms?
    Yes □ No □
  - If so, what are the different categories? (Identify by name)

Indigenous Treatment:

- What is the proposed treatment for the different mental health disorders?
- If certain herbs are used, what are they?
- Where are they obtained?
- How are they used?
- What are they intended to do to make the patient better?
- Typically, how long is the patient on treatment with herbs, etc.?

4. - How do you determine when a patient has been bewitched or has a calling?
  - Are there different treatment approaches for these? If so, identify these modalities of treatment.

5. - Do you follow up on patients? If so, how often?
  - What are the outcomes?

### Treatment for Schizophrenia

6. - Are you familiar with the mental health disorder termed schizophrenia? If yes, how do you diagnose it and what is the management plan?
  - When a patient has the following symptoms, what would your diagnosis be?
    i. Delusions
    ii. Conceptual disorganization
    iii. Hallucinations
    iv. Grandiosity
    v. Suspiciousness or persecution
    vi. Hostility
7. When a patient has the following symptoms, what would your diagnosis be?
  i. Asociality
  ii. Lack of drive and motivation outlined by apathy or avolition
  iii. Lack of enjoyment and social interactions
  iv. Anticipatory anhedonia and lack of facial expression
  v. Gesturing and speech output such as alogia or aprosody and blunting of affect
8. How do you treat or manage each symptom (negative symptoms of schizophrenia) mentioned in (c)?
9. If there is a medicinal concoction that is used to treat the symptoms mentioned in (c), what is the dosage and how is it measured?
10. What is the cost per treatment?

7. - Have you end up referred institutional care for a patient? If so how often?
  - What is your opinion on western medicine in managing mental health disorders?
  - Do you think traditional and western medicine can be used together? If so how?
  - How would you like to see traditional medicine used in communities more effectively?

### General

Any further comments?
